# Electroacupuncture Attenuates Cognitive Impairment in Rat Model of Chronic Cerebral Hypoperfusion via miR-137/NOX4 Axis

**DOI:** 10.1155/2021/8842022

**Published:** 2021-04-19

**Authors:** Xiaochen Bi, Yanfei Feng, Zemin Wu, Jianqiao Fang

**Affiliations:** ^1^School of Basic Medical Sciences, Zhejiang Chinese Medical University, Hangzhou, Zhejiang, China; ^2^Department of Urology, The Second Affiliated Hospital of Zhejiang Chinese Medical University (The Xinhua Hospital of Zhejiang Province), Zhejiang Provincial Key Laboratory of Traditional Chinese Medicine, Hangzhou, Zhejiang, China; ^3^The Third Affiliated Hospital of Zhejiang Chinese Medical University, Hangzhou, Zhejiang, China; ^4^Department of Neurobiology and Acupuncture Research, The Third Clinical College, Zhejiang Chinese Medical University, Key Laboratory of Acupuncture and Neurology of Zhejiang Province, Hangzhou, Zhejiang, China

## Abstract

Electroacupuncture has shown protective effects on cognitive decline. However, the underlying molecular mechanisms are not clear. The present study was conducted to determine whether the cognitive function was ameliorated in cerebral hypoperfusion rats following electroacupuncture and to investigate the role of miR-137/NOX4 axis. In this study, chronic cerebral hypoperfusion (CCH) model was established by bilateral common carotid artery occlusion. Electroacupuncture treatment attenuated brain injury in CCH model group via regulating miR-137/NOX4 axis. Furthermore, the data of neuronal apoptosis and oxidative stress were observed. Our findings indicated that (1) neuronal apoptosis and oxidative stress in CCH rats were significantly increased compared with control group; (2) the animal cognitive performance was evaluated using the Morris water maze (MWM). The results showed that electroacupuncture therapy ameliorated spatial learning and memory impairment in cerebral hypoperfusion rats; and (3) electroacupuncture therapy reduces neuronal apoptosis and oxidative stress by activating miR-137/NOX4 axis. These results suggest that electroacupuncture therapy for CCH may be mediated by miR-137/NOX4 axis. Electroacupuncture therapy may act as a potential therapeutic approach for chronic cerebral hypoperfusion.

## 1. Introduction

Adequate cerebral blood flow perfusion is the key factor to maintain normal brain function. Chronic cerebral hypoperfusion (CCH), also known as chronic cerebral ischemia, usually causes A*β* accumulation, high phosphorylation of Tau protein, neuroinflammation, oxidative stress, white matter damage, synaptic dysfunction, and excessive autophagy, which may lead to neuronal injury in vulnerable regions of the brain, such as the hippocampus and cerebral cortex, showing learning and memory decline. Increasing evidence suggests that CCH serves as a key vascular risk factor associated with Alzheimer's disease, vascular cognitive impairment, and cognitive impairment [1–3]. However, there is no effective treatment for CCH. Therefore, it is of great significance to study the treatment and mechanism of CCH.

Electroacupuncture is a method to prevent and cure diseases by combining acupuncture with electric stimulation and passing through the needle with a little electric current which is close to the bioelectricity of the human body. Electroacupuncture, which includes traditional acupuncture and modern electrotherapy technology, was frequently reported attenuating cognitive dysfunction in patients with stroke, Alzheimer's disease, and mild cognitive impairment [4–6]. It is worth mentioning that electroacupuncture has certain curative effect on CCH, which can regulate the hippocampus, reduce the brain damage of ischemic stroke rats, and improve cognitive dysfunction [2, 7–9]. It has been proved that electroacupuncture may modulate PKA/CREB signaling pathway to improve learning impairment and reduce hippocampus synaptic plasticity damage in rats with hypoperfusion [10]. Electroacupuncture is a common method to improve cognitive impairment. The salutary effect of electroacupuncture is related to the specific electrical stimulation parameters and acupoint selected, which can cause physiological metabolism regulation in order to treat chronic cerebral perfusion.

MicroRNAs are noncoding single-stranded RNA molecules that are involved in the regulation of gene expression of transcription and play an important role in a variety of metabolic activities [11]. It has been noted that various members of the miRNA family can regulate CCH-related physiological mechanisms through the regulation of target genes [12–14]. Ai et al. found that miR-195 targets CX3CL1/CX3CR1 to inhibit inflammation and alleviate neurological deficits [15].

miR-137 is an important member of this group, and loss of miR-137 can also lead to synaptic plasticity, behavioral duplication, learning, and impaired social behavior [12, 16–18]. At present, the mechanism of microRNAs in CCH has been paid more and more attention. In this study, we assessed the therapeutic effects of electroacupuncture in CCH rats and explored the underlying molecular mechanisms.

## 2. Materials and Methods

### 2.1. Animals

Male Sprague Dawley (SD) rats, 18–20 weeks old, weighing between 350 ± 50 g, were obtained from the Beijing SPF Biotechnology Co., Ltd. Animals were housed in a group of 5/cage at 25°C in a light-controlled (12 h light/dark cycle, 22–26°C) room, providing free access to water and food. All the experiment animal procedures were followed the Animal and Use Committee of Ethical Committee of Zhejiang Chinese Medical University (ZSLL-2016-85).

### 2.2. Cell Culture

The normal human embryonic kidney cells (HEK293) were acquired from American Type Culture Collection. HEK293 cells were cultured in DMEM medium with 10% fetal bovine serum (FBS, Gibco, USA) and 1% penicillin-streptomycin (Solarbio Science and Technology Co., China) at 37°C in a humidified atmosphere (5% CO_2_).

### 2.3. Luciferase Reporter Assays

For luciferase reporter assay, the wild-type psic-CHEC2-NOX4 and mutant psic-CHEC2-NOX4 were cotransfected with miR-137 into HEK293 cells. The synthetic NOX4 gene 3′-UTR was introduced into psic-CHEC-2, and the complementary mutation sites of the seed sequences were designed on the wild-type. The target fragments were inserted into psic-CHEC-2 vector by using T4 DNA ligase after being digested by restriction enzyme. Each group was transfected with psic-CHEC2-NOX4-mut and psic-CHEC2-NOX4-wt vectors by Lipofectamine 3000 (Invitrogen, San Diego, CA, USA) in antibiotic-free medium for 48 h. Luciferase activity was measured with the Dual-Luciferase® Reporter Assay System (Promega).

### 2.4. Induction of CCH and Electroacupuncture Treatment

The bilateral common carotid artery occlusion (BCCAO) model was established to simulate CCH. The rats were acclimatized for 1 week before the experiment and fasted for 12 h before surgery, during which period, water was withheld in the last 4 h. The rats were randomly split into control group, sham group, model group, drug group, and electroacupuncture group. Except the control group, the mice were treated with electroacupuncture 2 days after operation, once a day for 4 weeks. After the rats were fixed, “baihui point” and “dazhui point” were punctured by 0.30 mm × 0.30 mm acupuncture needles, and then the two points were connected by Korean electroacupuncture apparatus (1 mA, 2/15 Hz) for 30 minutes. The “baihui point” is located above the apex auriculate in the midline of the head. The “dazhui point” is located on the posterior midline and in the depression below the spinous process of the 7^th^ cervical vertebra in the prone position [19]. Both electroacupuncture group and CCH model group were performed bilateral common carotid artery ligation. Sham group had the same operation, except no artery ligation. Strict aseptic operation was maintained during the operation to prevent infection. The control group was treated with acupuncture and moxibustion only, but no current was connected. The drug group was treated with apocynin (Sigma-Aldrich, St. Louis, MO, USA) of 15 mg/kg/day and the drug control group was treated with normal saline.

### 2.5. Enzyme-Linked Immunosorbent Assay (ELISA)

The levels of NOX4, reactive oxygen species (ROS), superoxide dismutase (SOD), malondialdehyde (MDA), and catalase (CAT) were detected by ELISA. The experiment was carried out in accordance with the instructions of the kit, and the OD value of each hole at the specified wavelength was measured in sequence. The standard OD value is calculated according to the standard OD blank OD, and the standard curve is drawn with the standard concentration as the ordinate and the OD value as the abscissa. According to the results of OD blank OD, the concentration of the sample was calculated according to the standard curve.

### 2.6. qRT-PCR Assay

Total RNA was extracted using miRNeasy Mini Kit (Qiagen). U6 RNA served as an endogenous reference. The reactions were carried out in triplicate in an Applied Biosystems 7500 Fast Real-Time PCR System (ABI7500) in 20 *μ*L reaction mixtures. The relative expression of the target genes was detected by the method of 2^−ΔΔCT^.

### 2.7. Western Blot Analysis

*Extracting Proteins from the Brain.* Homogenized brain tissue was split in RIPA lysis buffer (Beyotime, Shanghai, China) supplemented with protease inhibitor (Roche, Basel, Switzerland). 20 mg of each protein preparation was electrophoretically separated on a 15% SDS-PAGE gel and transferred to a nitrocellulose membrane. The membrane was incubated with anti-eNOS and anti-iNOS antigen antibody (ViroGen Corporation, U.S.A., diluted at 1 : 1000) for 1 h at room temperature. Western blots were exposed to X-ray film with an enhanced chemiluminescence kit (BD Biosciences, U.S.A.). Finally, each area of protein band was detected and assessed by Image Lab™ Software (Bio-Rad). Each test was conducted in triplicate experiments.

### 2.8. TUNEL Assay

The number of apoptosis cells in rat brain was detected by in situ cell death test kit. The deparaffinized sections were cubated with protease K (10mmol/L) for 15 min, followed by 60 min with the TUNEL reaction mixture and then 30 min with converter-POD. TUNEL stained tissue sections were then photographed using light microscopy.

### 2.9. Statistic Processing

Data were analyzed and expressed as the mean ± standard deviation (SD) using SPSS 22.0. Comparison between every two groups was performed through *t* test. If *p* values were <0.05, differences were considered statistically significant.

## 3. Results

### 3.1. The Interaction between miR-137 and NOX4

**Figure 1:**
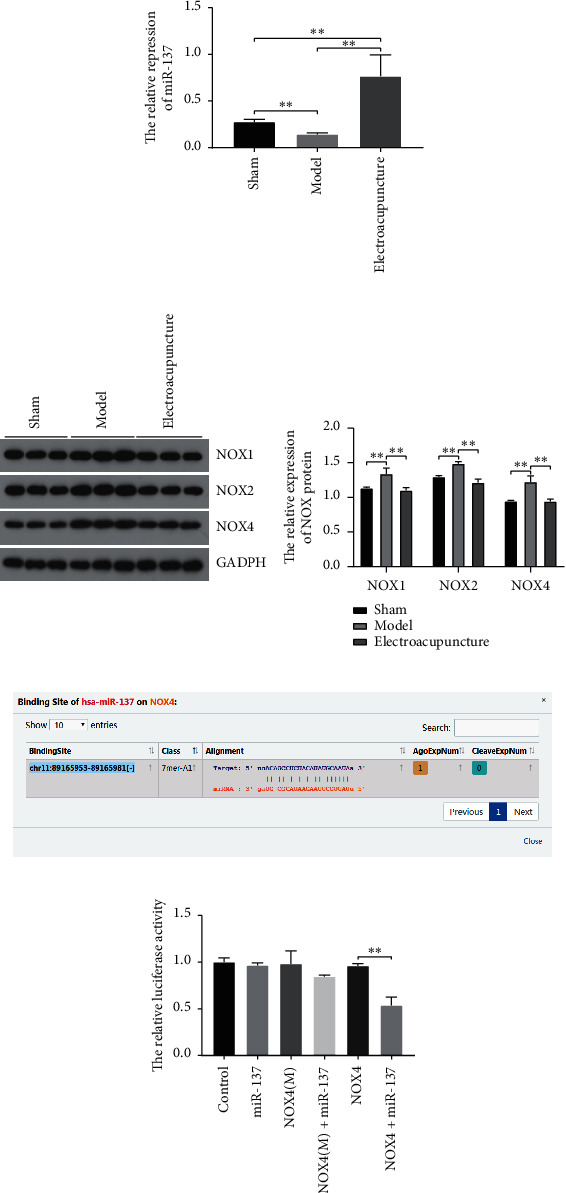
The interaction between miR-137 and NOX4. (a) The relative expression of miR-137 in the sham, model, and electroacupuncture groups, respectively. (b) The expression of NOX proteins in the sham, model, and electroacupuncture groups, respectively. (c) The interaction between miR-137 and NOX4 was predicted on ENCORI databases. (d) The interaction between miR-137 and NOX4 was detected by dual luciferase experiments. Data were normalized to the control and presented as mean ± SD, ^*∗∗*^*p* < 0.01.

To investigate the effect of electroacupuncture on miR-137, we examined the expression of miR-137 by qRT-PCR. The results showed that the expression level of miR-137 was significantly decreased in the CCH model group compared with the control and sham groups and treated with electroacupuncture, miR-137 expression significantly upregulated (Figure 1(a)).

The expressions of NOX1, NOX2, and NOX4 were detected by western blot. The results suggested that the protein expressions of NOX1, NOX2, and NOX4 were upregulated and treated with electroacupuncture, the expression significantly downregulated (Figure 1(b)).

In order to figure out the relation between miR-137 and NOX, we predicted the target genes of miR-137 on ENCORI databases. The results showed that the seed sequence of miR-137 could perfectly match the 3′-UTR position of NOX4 mRNA sequence, which suggested NOX4 was a potential target of miR-137 (Figure 1(c)).

To further confirm whether miR-137 directly targets NOX4, the recombinant reporter vectors were cotransfected with miR-137. The results showed that the luciferase activities of miR-137 group, NOX4 group, NOX4(m) group, and NOX4(m) + miR-137 group had no significant difference compared with the control group, while the activity of NOX4 + miR-137 group was remarkably lower than the control group (Figure 1(d)).

### 3.2. Electroacupuncture Therapy Improved CCH Model Spatial Learning and Memory Ability

The potential protective effects of electroacupuncture against spatial learning and memory deficits of CCH-induced rats were assessed using the MWM test. The results showed that the model group and electroacupuncture control, drug control, and miR-137 control groups had significantly longer escape latency from the 2nd to the 5th time during training compared with the sham group. Analysis of escape latencies showed a significant reduction in of the electroacupuncture group, drug group, and miR-137 group. The times of crossing the platform in each group were counted. On the fifth day of treatment, the results showed that the time of crossing the platform in the model and its control groups was significantly reduced. The times of crossing the platform increased significantly in the electroacupuncture group, drug group, and miR-137 group compared with the control group (Table 1).

In addition, we also counted the number of rats passing through the platform in each group. The results showed that the number of rats in the model group and electroacupuncture control group, drug control group , and miR-137 control groups passed through the platform was significantly decreased in the control and sham groups. Compared to its control groups, the number of rats passing the platform increased significantly in the electroacupuncture, drug, and miR-137 groups (Table 2). MWM test suggested that electroacupuncture therapy improved CCH model rat spatial learning and memory ability.

### 3.3. Electroacupuncture Therapy Inhibited Neuronal Apoptosis

**Figure 2:**
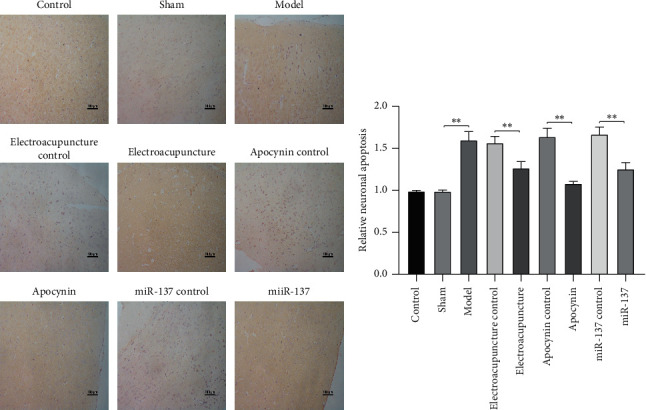
Electroacupuncture therapy inhibited neuronal apoptosis. The number of apoptosis cells in rat brain was detected by TUNEL.

The number of TUNEL-positive cells (brown-yellow cells) in the model group and electroacupuncture control group, drug control group, miR-137 control group was markedly larger than that in the control group and sham group, while the positive cells in electroacupuncture group, drug group, and miR-137 group were significantly decreased than the control group (Figure 2).

**Figure 3:**
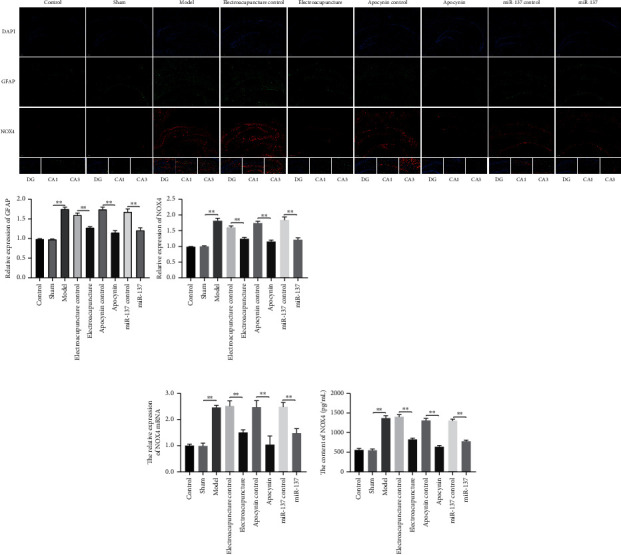
The expression of GFAP and NOX4. (a) The expression of glial fibrillary acidic protein (GFAP) (green) and NOX4 (red) in the hippocampus of rats by immunofluorescence. (b) The relative expression of NOX4 mRNA by qRT-PCR. (c) The content of NOX4 was detected by ELISA. The error bars are presented as mean ± SD, ^*∗∗*^*p* < 0.01.

We investigated the expression of glial fibrillary acidic protein (GFAP) in the hippocampus of rats by immunofluorescence. The results showed that the protein expression of GFAP was upregulated in model group, and the expression significantly downregulated treated with electroacupuncture, drug, and miR-137 (Figure 3(a)).

Both TUNEL-positive cells and GFAP expressions revealed that electroacupuncture may inhibit neuronal apoptosis.

### 3.4. Electroacupuncture Therapy Downregulated the Expression of NOX4

In order to confirm that electroacupuncture therapy is improving spatial learning and memory deficits in CCH rats via miR-137/NOX4 axis. We detected the expression of NOX4 by qRT-PCR and immunofluorescence. Compared with control and sham groups, the expression of NOX4 was significantly increased in model group. After electroacupuncture and miR-137 treatment, the expressions of NOX4 were significantly decreased, which was consistent with the results of drug intervention (Figures 3(a) and 3(b)). To further validate our results, we performed ELISA experiments. The content of NOX4 protein detected by ELISA was consistent with the above results (Figure 3(c)).

### 3.5. Electroacupuncture Therapy Reduced the Expression of NOS

**Figure 4:**
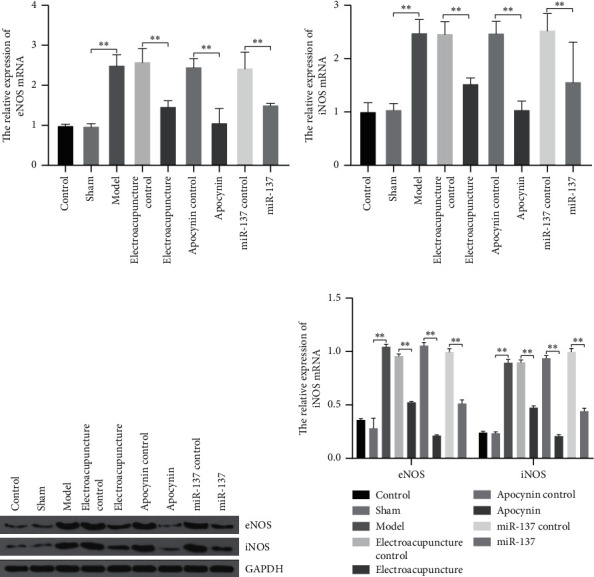
The expression of eNOS and iNOS. (a) The relative expression of eNOS mRNA. (b) The relative expression of iNOS mRNA. (c) The expression of eNOS and iNOS protein. At least three repeats were carried out, and the mean ± SD is presented, ^*∗∗*^*p* < 0.01.

In addition, we also studied the effects of electroacupuncture on oxidative stress. qRT-PCR and western blot were performed to examine the expression of endothelial nitric oxide synthase (eNOS) and inducible nitric oxide synthase (iNOS). The expressions of eNOS and iNOS in the model, electroacupuncture control, drug control, and miR-137 control groups were significantly higher than that in control and sham groups. After electroacupuncture and miR-137 treatment, the expressions of eNOS and iNOS were significantly decreased, which was consistent with the results of drug intervention (Figures 4(a)–4(c)).

### 3.6. Electroacupuncture Therapy Reduced the Damage Caused by Oxidative Stress

**Figure 5:**
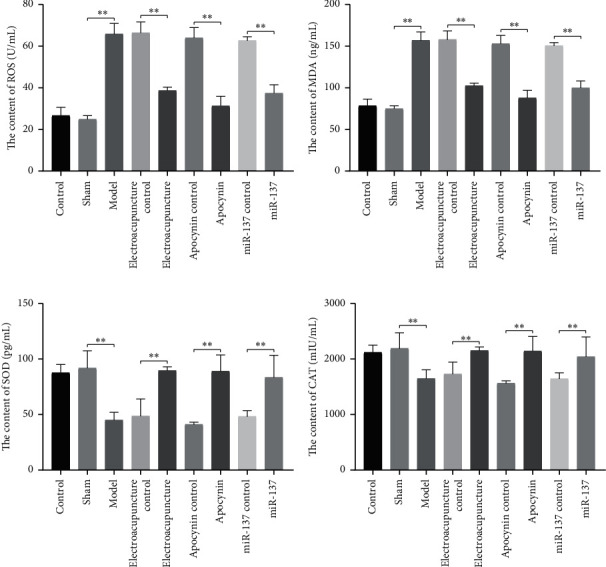
The oxidative stress in rat. (a) The content of ROS. (b) The content of MDA. (c) The content of SOD. (d) The relative expression of CAT. ROS, MDA, SOD, and CAT were detected by ELISA. At least three repeats were carried out, and the mean ± SD is presented, ∗∗*p* < 0.01.

In order to further verify our conclusion, we were using ELISA to detect some oxidative stress indicators, such as ROS and MDA. As illustrated in Figures 5(a) and 5(b), the contents of ROS and MDA were obviously increased in the model group compared with control and sham groups. After electroacupuncture therapy, drug, and miR-137 intervention, the contents of ROS and MDA were significantly decreased.

Compared with control and sham groups, the contents of SOD and CAT in model group were significantly reduced, and after electroacupuncture therapy and miR-137 intervention, the contents of SOD and CAT were significantly increased in line with the positive drug intervention (Figures 5(c) and 5(d)).

## 4. Discussion

Due to an increase in the aging population, age-related cognitive impairment, in particular vascular cognitive impairment, becomes increasingly challenging worldwide, without effective medications. CCH is caused by various reasons, which easily lead to cerebral ischemia and hypoxia, and even progressive cognitive dysfunction [2]. The aim of this study was tantamount to elucidate the effects of electroacupuncture on the brain of CCH rats and its molecular mechanism.

In order to investigate the role of miR-137 in the treatment of CCH rats with electroacupuncture, MWM task was used to assess spatial learning and reference memory of the rats. Apocynin, as a positive control, is an inhibitor of NOX, which used to intervene CCH [14]. The results showed that the number of rats crossing the platform significantly increased after electroacupuncture and miR-137 intervention; the trend is consistent with the results of positive drug intervention. It indicated that electroacupuncture could effectively improve the rats' cognitive function by upregulating the expression of miR-137.

The apoptosis of hippocampal neurons is the critical factor to vascular dementia induced by CCH, which lead to cognitive impairment [7, 20]. Hence, it is very important to suppress the apoptosis of neurons in the process of CCH prevention. The results of TUNEL showed that the apoptosis rate of model rats was significantly higher than that of control group. Electroacupuncture and miR-137 can decrease the apoptosis rate of CCH model. GFAP serves as a marker of astrocytes activation. Astrocytes are the most widely distributed cells in brain, which have a protective effect on neurons [21–23]. We detected the expression of GFAP. The results showed that the expression of GFAP significantly upregulated in CCH model. However, the expression of GFAP downregulated when treated with electroacupuncture and miR-137 intervention. It indicated that electroacupuncture could inhibit the neuronal apoptosis by regulating miR-137.

Oxidative stress plays an important role in the development of cognitive impairment that is caused by CCH-induced pathology. The family of nicotinamide adenine dinucleotide phosphate (NADPH) oxidases (NOXs), which are enzyme complexes that transport electrons across the membrane and generate superoxide, plays a major role in generating ROS. NOX is an important source of ROS in blood vessels. It has been reported that vascular functional and structural changes are attributable to NOX-driven intravascular ROS generation [24]. The injury of cerebral vessels was mainly initiated by NOX1, NOX2, and NOX4 [25, 26]. Our study suggested the interaction between miR-137 and NOX4. Further research found that the content and expression of NOX4 were significantly higher in the CCH model group than control group. After electroacupuncture therapy and miR-137 intervention, the content and expression of NOX4 were markedly decreased. It is speculated that electroacupuncture therapy inhibited NOX4 and alleviated the oxidative stress. In addition, we detected the expression of NOX4 in each group of rats by immunofluorescence. The results showed that NOX4 immunofluorescence staining was localized in the cytoplasm. Compared with the control group, the fluorescence intensity of NOX4 in the CCH model group was significantly increased but decreased after electroacupuncture treatment and miR-137 intervention. The results showed that CCH could induce oxidative stress, while electroacupuncture could decrease the expression of NOX4 and oxidative stress by regulating miR-137, which partly alleviates the injury caused by CCH.

In addition, we examined the effect of CCH on the endogenous oxidative stress-related indices, as an index of eNOS, iNOS, ROS level, MDA level, SOD activity, and CAT activity in the rat striatum in order to clarify the role of electroacupuncture on CCH-induced oxidative stress. SOD and CAT are important antioxidant enzymes, which can alleviate the injury caused by oxidative stress, and the levels of ROS and MDA reflect the degree of lipid peroxidation in cells and tissues [27–29]. The results showed that electroacupuncture could reduce the levels of ROS and MDA in serum. The content of SOD and CAT in electroacupuncture group was significantly higher than that in model group. On the one hand, nitric oxide relaxes the vascular smooth muscle and relaxes blood vessels. Nitric oxide, on the other hand, can interact with ROS to exacerbate the damage brought about by cerebral ischemia [20]. It is noteworthy that electroacupuncture therapy can significantly reduce the expression of eNOS and the production of iNOS. The reason may be that electroacupuncture therapy can upregulate the expression of miR-137, which results in the inhibition of NOX4 expression, and oxidative stress was suppressed, and cognitive function was improved. These results suggest that electroacupuncture treatment could improve cerebral ischemia after CCH via miR-137/NOX4 signaling pathway and subsequent antioxidant defense activation.

## 5. Conclusion

Taken together, our data indicated that electroacupuncture could inhibit neuronal apoptosis, ameliorate learning deficits, and alleviate brain impairment of cerebral hypoperfusion rats, with a probable regulation of miR-137/NOX4 axis.

## Figures and Tables

**Table 1:** The escape latency of rats in each group in the Morris water maze (MWM).

Group	1^st^ day	2^nd^ day	3^rd^ day	4^th^ day
Control	34.54 ± 2.46	29.71 ± 2.11	21.98 ± 1.57	13.28 ± 2.03
Sham	33.51 ± 2.42	28.82 ± 2.08	21.32 ± 1.54	14.50 ± 1.05
Model	82.78 ± 8.35^*∗∗*^	73.20 ± 3.78^*∗∗*^	54.16 ± 2.80^*∗∗*^	36.83 ± 1.90^*∗∗*^
Electroacupuncture control	85.36 ± 4.40	73.41 ± 3.79	54.32 ± 2.80	36.94 ± 1.90
Electroacupuncture	51.40 ± 3.08^*∗∗*^	44.20 ± 2.65^*∗∗*^	32.71 ± 1.96^*∗∗*^	22.24 ± 1.33^*∗∗*^
Apocynin control	85.57 ± 4.41	73.60 ± 3.79	55.79 ± 4.03	37.03 ± 1.91
Apocynin	34.16 ± 2.45^*∗∗*^	29.37 ± 2.10^*∗∗*^	21.74 ± 1.56^*∗∗*^	14.78 ± 1.06^*∗∗*^
miR-137 control	83.57 ± 4.33	71.87 ± 3.72	53.18 ± 2.76	36.16 ± 1.87
miR-137	49.80 ± 3.02^*∗∗*^	42.83 ± 2.60^*∗∗*^	31.69 ± 1.92^*∗∗*^	22.55 ± 2.27^*∗∗*^

Data were normalized to the control and are presented as mean ± SD, ^*∗∗*^*p* < 0.01.

**Table 2:** The number of passes through platform of rats in each group from the 2^nd^ to the 5^th^ time during training.

Group	Number
Control	5.33 ± 0.58
Sham	5 ± 1.00
Model	1.67 ± 0.58^*∗∗*^
Electroacupuncture control	1.33 ± 0.58
Electroacupuncture	4.33 ± 0.58^*∗∗*^
Apocynin control	1.67 ± 0.58
Apocynin	5 ± 1.00^*∗∗*^
miR-137 control	1.67 ± 0.58
miR-137	4.33 ± 0.58^*∗∗*^

Data were normalized to the control and are presented as mean ± SD, ^*∗∗*^*P* < 0.01.

## Data Availability

The data used to support the findings of this study are available from the corresponding author upon request.
